# Exploring the Influence of Small-Scale Geographical and Seasonal Variations Over the Microbial Diversity in a Poly-extreme Athalosaline Wetland

**DOI:** 10.1007/s00284-023-03395-w

**Published:** 2023-07-25

**Authors:** Coral Pardo-Esté, Sergio Guajardo Leiva, Francisco Remonsellez, Eduardo Castro-Nallar, Juan Castro-Severyn, Claudia P. Saavedra

**Affiliations:** 1 Laboratorio de Microbiología Molecular, Facultad de Ciencias de la Vida, Universidad Andres Bello, Santiago, Chile; 2 Departamento de Microbiología, Facultad de Ciencias de la Salud, Universidad de Talca, Campus Talca, Avda. Lircay s/n, Talca, Chile; 3 Centro de Ecología Integrativa, Universidad de Talca, Campus Talca, Avda. Lircay s/n, Talca, Chile; 4 Laboratorio de Microbiología Aplicada y Extremófilos, Departamento de Ingeniería Química, Universidad Católica del Norte, Antofagasta, Chile; 5 Centro de Investigación Tecnológica del Agua en el Desierto-CEITSAZA, Universidad Católica del Norte, Antofagasta, Chile; 6 Departamento de Ciencias Farmacéuticas, Facultad de Ciencias, Universidad Católica del Norte, Antofagasta, Chile

## Abstract

Microorganisms are the most diverse life form on the planet and are critical for maintaining the geochemical cycles, especially in extreme environments. Bacterial communities are dynamic and respond directly to changes in abiotic conditions; among these communities, poly-extremophiles are particularly sensitive to perturbations due to their high specialization. Salar de Huasco is a high-altitude wetland located on the Chilean Altiplano exhibiting several conditions considered extreme for life, including negative water balance, extreme variations in temperature and pH values, high UV radiation, and the presence of various toxic metal(oids). However, previous reports have revealed a diverse bacterial community that has adapted to these conditions, here, we aimed to determine whether microbial community diversity and composition changed in response to geographical and seasonal variations. We found that there are significant differences in diversity, abundance, and composition in bacterial taxa that could be attributed to local geographical and seasonal variations, which in turn, can be associated with microbial traits. In conclusion, in this poly-extreme environment, small-scale changes can trigger significant changes in the microbial communities that maintain basic biogeochemical cycles. Further in depth analysis of microbial functionality and geo-ecological dynamics are necessary to better understand the relationships between seasonal changes and bacterial communities.

**Supplementary Information:**

The online version contains supplementary material available at 10.1007/s00284-023-03395-w.

## Introduction

Extreme environments can be used as natural laboratories to study the dynamics that occur between microbial life and the geochemical conditions, given that microorganisms are one of the main engines maintaining critical cycles [1]. These microbial communities require a certain level of specialization to thrive in these conditions, yet spatial diversity is critical to maintain life when several stressors co-occur [2].

Salar de Huasco, our case study, is a protected athalosaline wetland located in the Chilean Altiplano (20° 18′ 18″ S; 68° 50′ 22″ W) situated at 3800 masl [3, 4]. This Salar is considered a poly-extreme environment because of several co-occurring stressors, including high solar radiation and salinity, negative water balance, extreme variations in temperature, pH values -ranging from neutral to alkaline-, and low atmospheric pressure, and given its geological origin, this wetland is rich in sulfur, copper, gold, arsenic, among many other metal(oids) [3].

In the Altiplano plateau, changes between the Winter and Summer seasons are significant, there is a well-marked wet/dry period; also, its geographical conformation and elevation (3700–4400 masl) causes distinctive meteorological conditions reflected particularly in water availability, as rainfall in this area is restricted to the austral summer season (November to March) linked to the South American Summer Monsoon. However rainy days tend to cluster and are separated by dry episodes of similar time lengths, and a defined hydrometeorological cyclicity resulting in a complex, variable, and spatially heterogeneous wetland [5, 6].

Previous studies of microbial communities in water and shallow sediments have determined that there is an important level of variation within a relatively small area in Salar de Huasco, which is reflected in a highly heterogeneous microbial community including several unknown taxa, e.g., [7–11]. Moreover, several studies have been carried out in the area that have contributed to understanding the role, functionality, and resistance mechanisms of microbial communities in element cycling within Salar de Huasco [8, 12].

Seasonal changes, associated with water availability and temperature, are linked to variations in the structure and composition of microbial communities, as they are not isolated from the surrounding environments [13, 14]. Furthermore, recently it was determined that there is cyclicity in the wet and dry seasons in Salar de Huasco, which was associated with changes in the microbial communities [6]. Therefore, in this study we set out to address whether the composition of the bacterial community changes in response to small-scale temporal and geographical variations in this environment to further characterize the dynamics of this extreme but fragile ecosystem that is maintained by a delicate balance in ecological functions lead by microorganisms, that are part of the biological patrimony that should be conserved.

## Materials and Methods

### Field Trips and Sample Collection

Subsurface sediment samples were collected at 4 different sites (H0, H1, H3, and H4) in the Salar de Huasco (SH) (Supplementary Fig. S1) during two seasons: the summer of 2017 and the winter of 2018 (in duplicate). Samples were taken to a depth of 5 cm and approximately 1 m inward from the main lagoon shoreline. These were collected in sterile 50 ml tubes and kept in a cooler for transport to the laboratory, where they were stored at − 20 °C until processing.

### Physicochemical Parameters

At each site, temperature, salinity, and pH (HI 98192 and HI 2211, HANNA Instruments) were recorded in situ. Total arsenic content was later determined through an ELAN DRC-e ICP-MS (PerkinElmer®) at INQUISAL-CONICET (San Luis, Argentina), following ASTM “American Society for Testing and Materials” standard methods (TMECC: 04.12-B and 04.14).

### DNA Extraction and Sequencing

Total DNA was extracted from the sediment samples from each SH site using the DNeasy PowerSoil kit (Qiagen Inc., Hilden, Germany) according to the manufacturer’s instructions using 0.25 g of input material. DNA integrity, quality, and quantity were verified by 1% agarose gel electrophoresis, OD_260/280_ ratio spectroscopy, and fluorescence using a Qubit 3.0 fluorometer along with the Qubit dsDNA HS assay kit (Thermo Fisher Scientific, MA, USA). Next, DNA samples were sent to the Environmental Sample Preparation and Sequencing Facility at the Argonne National Laboratory (Illinois—USA) for amplification of the bacterial 16S rRNA gene V4 region (~ 250 bp) using the 515F and 806R primers [15], construction of 250 bp paired-end libraries and sequencing on a MiSeq (Illumina) platform.

### Taxonomic Composition Analysis

This analysis was conducted in R v4.0.3 and RStudio v1.3.1093 following the DADA2 v1.16.0 R package pipeline [16], in order to infer amplicon sequence variants (ASVs) present in each sample. Briefly, the reads were evaluated for quality control and subsequently trimmed (Ns = 0, length ≥ 150 bp, expected errors ≤ 2), followed by dereplication, denoising, and merging of paired reads. Subsequently, the ASV table was built allowing a maximum of two expected errors, the chimeras were removed, and taxonomic assignment was carried out against the Silva v138 [17] database. ASVs identified as Eukarya, Chloroplast, and Mitochondria were removed. Moreover, a multi-sequence alignment was created to infer phylogeny using FastTree v2.1.10 [18]. Furthermore, a phyloseq-object (containing the ASVs, taxonomy assignment, phylogenetic tree, and the samples meta-data) was created using the R package Phyloseq v1.34.0 [19]. Finally, taxonomy composition and relative abundance plots were generated using the ggplot2 v3.3.3 and ampvis v2.7.4 [20] R packages. The differential abundance of microorganisms between seasons was analyzed with DESeq2 v1.40.1 R package [21], normalizing through Variance Stabilizing Transformation and performing the Wald Test.

### Alpha-Diversity Analysis

Alpha diversity metrics corrected by unobserved species (Shannon and Simpson indexes) were calculated using the R packages breakaway and DivNet [22, 23]. Both diversity indexes were used to calculate the Effective Number of Species (ENS) by the exponential transformation of the Shannon index and reciprocal transformation of the Simpson index. The normality of the alpha diversity (ENS) distribution was tested using a Shapiro–Wilk test implemented in the shapiro.test function in R. To test the statistical significance of alpha diversity (Shannon and Simpson) across predictors variables (season by site, Arsenic concentration, Salinity, and pH), we used an Analysis of variance (ANOVA) implemented in R (aov function in stats package) and pairwise comparisons with Tukey's HDS (TukeyHSD function in stats package). We fitted a generalized linear model implemented in R (glm function in stats package) with a gaussian distribution to model the alpha diversity variation between seasons by sites, Arsenic concentration, Salinity, and pH values. We used the Visreg package in R to visualize the generalized linear model. The statistical significance of the models was tested using an ANOVA implemented in R (anova.glm function in the stats package).

### Beta-Diversity Analysis

Bray–Curtis (corrected by unobserved species) and weighted UniFrac metrics were calculated using the DivNet [22] and phyloseq [19] packages in R. To identify drivers of beta diversity, we tested whether the dispersion among groups was homogeneous using the function betadisper implemented in the Vegan package [24]. Later, we assessed the statistical significance of the beta diversity among season by site, Arsenic concentration, Salinity, and pH values using a Permutational Multivariate Analysis Of Variance (PERMANOVA) and pairwise comparisons with pairwise PERMANOVA implemented in the adonis and pairwise.adonis functions in Vegan [24] and pairwise Adonis packages, respectively. We did a constrained ordination analysis to extract and summarize the sample variation that the site variable can explain. To decide whether to apply a linear or a unimodal ordination method, we performed a Detrended Correspondence Analysis (DCA) using the decorana function in the Vegan package [24]. Then, we carried out a redundancy analysis (RDAs) using the Hellinger-transformed Bray–Curtis and weighted UniFrac distances based on the ASV abundance matrix in Ampvis2 [20]. The statistical significance of the selected variables was measured by an ANOVA test, and the statistical significance of the RDAs was tested using 9999 permutations.

### Biomarker Species

Were determined for each sampling site by performing indicator species analyses using the indicspecies package [25] in R. All ASVs present in only one sample were filtered from the ASV abundance table before the analysis. Biomarkers were filtered by their Indicator Value (IndVal = 1) index, which measures the association between a species and a site group. The statistical significance of this relationship was tested using a permutation test, keeping biomarkers with a *P*-value ≤ 0.01. A Boxplot of the indicator species' relative abundance (log-transformed) was plotted using the amp_boxplot function in the ampvis2 R package.

### Metabolic Pathways

Were inferred in association with the indicator species, using a phylogenetic placement approach implemented in the PAthway PRediction by phylogenetIC placement (paprica) software [26]. Briefly, reads corresponding to the indicator species (ASVs) were used to determine phylogenetic placements and select core genomes. Metabolic pathways were predicted for each core genome and read placement abundance data were used to generate an abundance matrix of pathways for each sample. The relative abundance (log-transformed) of the metabolic pathways from the indicator species by site were plotted in a heatmap using the pheatmap package in R.

## Results

In this study, we processed 2,391,048 paired reads and obtained a total of 7909 ASVs for further analysis. Our results of measured physicochemical parameters indicate variations in each sample site (Table 1, [27]) therefore we determined if these differences were associated only to small-scale geographical sites or other variables such as seasonal cyclicity were involved in changing the microbial diversity and/or composition.

**Table 1:** Physicochemical parameters measured in sediments from Salar de Huasco. Values are given as the mean of two replicates

Site	Summer				Winter			
	Salinity (%)	Conductivity (mS)	As (mg/kg)	pH	Salinity (%)	Conductivity (mS)	As (mg/kg)	pH
H0	11.9	6.082	9	8.8	1.5	0.752	26.09	7.6
H1	8.1	4.076	16.3	9.4	1.3	0.692	28.01	8.7
H3	2.2	1.122	49.2	8.5	118.3	60.13	69.19	8.4
H4	77.2	38.2	155	8.4	135.3	68.5	174.26	8.8

**Fig. 1:**
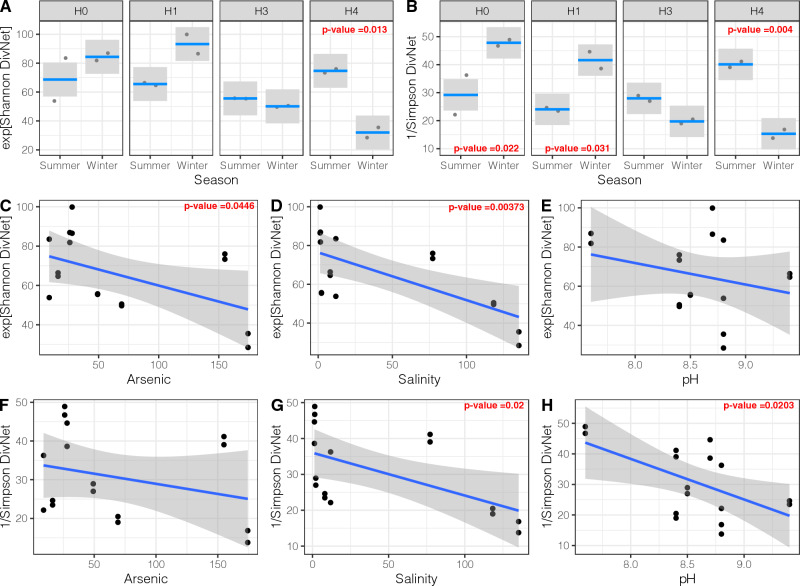
Generalized linear models (GLMs) of microbial alpha diversity (effective number of species based on Shannon and Simpson indexes) across different predictors. **a**, **b** GLMs of microbial alpha diversity between seasons by sites. **c**, **f** GLMs of microbial alpha diversity across arsenic concentrations. **d**, **g** GLMs of microbial alpha diversity across Salinity. **e**, **h** GLMs of microbial alpha diversity across pH. Smooth areas represent the confidence bands, dots represent the partial residuals, and lines the predicted values inferred from the models. Statistically significant differences in alpha diversity across predictors in the model (ANOVA or Tukey HSD, *P*-values) are signaled in red letters inside the plot (Color figure online)

To model the variation of alpha diversity across different predictors in the bacterial communities of SH, we constructed GLMs using a gaussian distribution since ENS (based on Shannon and Simpson indexes) has a normal distribution (Shapiro–Wilk, *P*-value > 0.05, Fig. 1). Using this approach, we found statistically significant differences in the alpha diversity of microbial communities between summer and winter seasons at specific sites, i.e., Shannon and Simpson’s indexes showed a significant difference between seasons in site H4 (Fig. 1a and b). On the other hand, only the Simpson index displayed a significant seasonal variation in sites H0 and H1 (Fig. 1b). Furthermore, in sites H0 and H1, the ENS was significantly higher (TukeyHSD, *P*-value < 0.05) in the winter samples than in the summer samples. Conversely, in site H4, the ENS was significantly higher (TukeyHSD, *P*-value < 0.05) in the summer samples than in the winter samples (Fig. 1a and b).

Likewise, we found statistically significant differences (ANOVA, *P*-value < 0.05) in the alpha diversity of microbial communities across Arsenic, salinity (NaCl), and pH values gradients (Fig. 1c–h). For all these environmental variables, we found that the ENS decreased as the indicators (i.e., arsenic, salinity –NaCl–, and pH) increased. Taken together, these results suggest that the season (at specific sites), as well as arsenic concentration, salinity, and pH, are good predictors of microbial diversity in the Salar de Huasco.

To determine whether microbial communities were homogeneous or presented a high turnover among seasons by sites, arsenic concentrations, salinity, and pH values, we calculated two beta-diversity indices (Bray–Curtis with unobserved species correction and weighted Unifrac). Since our data corroborated the hypothesis of homogeneity of variance (ANOVA, *P*-value > 0.05), we used a Permutational Multivariate Analysis of Variance (PERMANOVA) to test the differences in composition between samples. We found statistically significant differences (PERMANOVA, *P*-value ≤ 0.05) between beta diversity (Bray–Curtis and weighted Unifrac) of microbial communities from different sites. The “Site” variable explained 47.75–55.55% of the variance between samples when weighted Unifrac or Bray–Curtis were used, indicating that the geographical point in which the sample was taken could explain the variability. Pairwise comparisons between sites showed statistically significant differences in microbial beta diversity (Bray–Curtis and weighted Unifrac) between H0 v/s H3 and H1 v/s H3 (pairwise-PERMANOVA, *P*-value ≤ 0.05). Additionally, when the Bray–Curtis distance was used, we found statistically significant differences between H0 v/s H4 and H1 v/s H4. These results suggest that bacterial species' turnover significantly differed among sites, especially between the pairs H0–H1 v/s H3–H4.

**Fig. 2:**
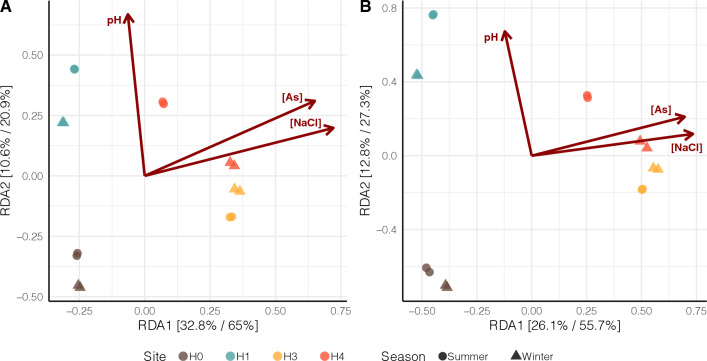
Beta-diversity analysis of microbial communities. **a** Redundancy analysis of Hellinger transformed Bray–Curtis distance (corrected by unobserved species). **b** Redundancy Analysis of Hellinger transformed weighted-UniFrac distance. The site was statistically chosen to constrain the multivariate space in a supervised approach. Each axis in the graph shows the percentage of variance explained in an unsupervised and supervised analysis

To further explore the bacterial community structure in SH, we performed a constrained ordination analysis, extracting and summarizing the maximum variation of the microbial composition explained by the sampling site (the constraint variable). Using a Detrended Correspondence Analysis (DCA), we determined the suitability of a linear ordination method (first DCA axis < 3), performing Redundancy analysis (RDA), showing that the constrained space explained 50.5% (Bray–Curtis) and 43.7% (weighted Unifrac) of the total beta-diversity variance (Fig. 2). The RDA analysis emphasizes the relevance of supplementary variables such (i.e., arsenic concentration, salinity, and pH) as the factors behind the variation extracted by the ordination axis. Consequently, the *X* axis would separate the samples according to their arsenic concentration and salinity, while the *Y* axis would do so according to pH.

Once we determined changes in diversity occur in response to small-scale environmental changes and seasonal variations, we aimed to determine variations in the structure and composition of the bacterial community. Overall, bacterial composition in summer and winter is dominated by Proteobacteria and Bacteriodota (Supplementary Fig. S2). However, there are notable changes in composition associated with the season and geographical site (Wald Test: *P* ≤ 0.01). Conversely the abundance of Acidobacteria and Planctomycetota is significantly reduced during winter, this is more noticeable in less abundant taxa. Particularly, in site H0 during winter, Cyanobacteria increases in abundance (Wald Test: *P* ≤ 0.01), and in site H1 a greater variety of taxa were detected. Moreover, H3 is less dynamic, as observed in the alpha diversity indexes. Finally, in H4, a marked increase of Bacteriodota during winter was observed (Wald Test: *P* ≤ 0.01), while in summer a greater overall diversity was found.

**Fig. 3:**
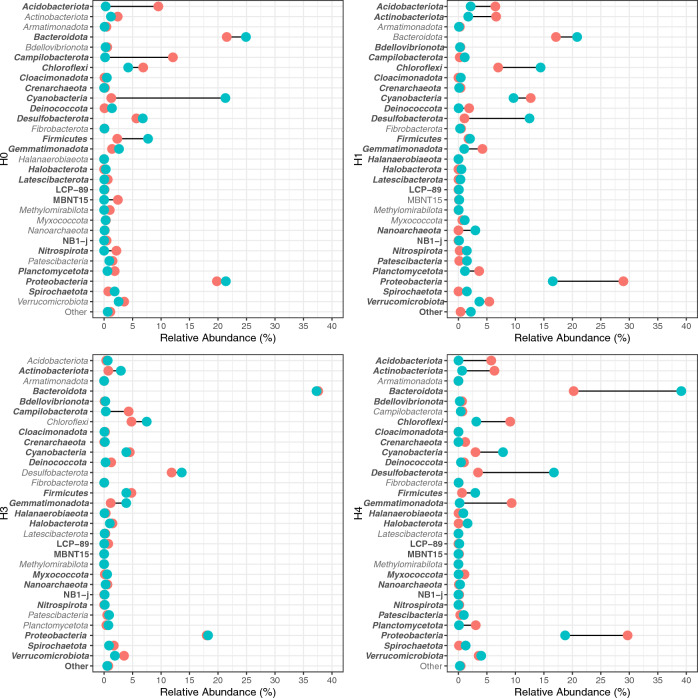
Seasonal changes in bacterial composition and abundance. Dumbbell charts indicate the relative abundances of the top 30 taxa (less common ASVs were grouped in “other”). Green dots indicate samples taken in winter and red dots indicate samples taken in summer. Phyla with significantly different abundance between seasons are displayed in bold (Wald Test: *P* ≤ 0.01) (Color figure online)

To characterize the dynamics of each taxon inter-seasonally, a dumbbell chart that illustrates changes in abundance in the top 30 taxa at the phylum rank was generated (Fig. 3), showing the differences in abundance between each site during the cycle of the seasons and the statistically significance of these differences (Fig. 3, Supplementary Table S1). For instance, in site H0 the Phyla that vary are Acidobacteriota, Campilobacterota, and Cyanobacteria (Wald Test: *P* ≤ 0.01). On the other hand, the H1 site shows the most variation in Desulfobacterota and Proteobacteria (Wald Test: *P* ≤ 0.01)*.* In H3 Campilobacterota and Gemmatimonadota displayed a higher variation in abundance between the seasons compared to the other phyla (Wald Test: *P* ≤ 0.01). Finally, for H4 Bacteriodota and Desulfobacterota are the taxa with most differences in abundance found by these analyses (Wald Test: *P* ≤ 0.01). Suggesting a variation in the structure of the community associated with changes in seasonality within a small geographical area (3785 m).

**Fig. 4:**
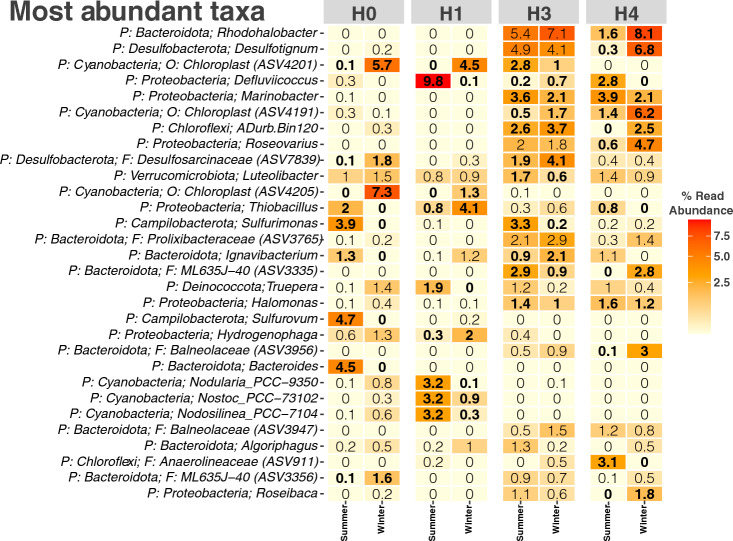
Most abundant taxa in sediments of Salar de Huasco. The heatmap shows the 30 most abundant ASVs in sites H0, H1, H3, and H4 in summer and winter, at ‘phylum’ (P) rank plus the lowest available rank; ‘class’ (C), ‘order’ (O), or ‘family’ (F) are indicated when no genus-level (G) taxonomic assignment was available. Taxa with significantly different abundance between seasons are displayed in bold (Wald Test: *P* ≤ 0.05)

Next, to further elucidate inter-seasonal variation in the bacterial community within the Salar, the most abundant taxa at each sampled site (at the lowest taxonomic rank available) were determined (Fig. 4). As expected, the bacterial communities are overall dominated by Bacteriodota and Proteobacteria members. However, some taxa that were very abundant at some sites were completely absent in others, namely: Cyanobacteria, *Sulfiromonas, Defluviicoccus,* and *Chloroflexi,* supporting the hypothesis of high variance adaptability to niche-specific conditions.

The results indicate that in site H0, major differences in abundances in the phylum Cyanobacteria (0% abundance in summer–7.3% abundance in winter)*,* family Desulfosarcinaceae (0.1–1.8%), and genus *Truepera* (0.1–1.4%) were found to be more common in winter, while organisms belonging to the genera *Sulfurimonas* (3.9–0%)*, Sulfurovum* (4.7–0%), and *Bacteroides* (4.5–0%) were more abundant in the summertime. Key players in site H1 include some unknown genus from Cyanobacteria (0–4.5%) and bacteria identified as *Thiobacillus* (0.8–4.1%) in winter, while organisms classified as *Defluviicoccus* (9.8–0.1%) as well as some Cyanobacteria (*Nostoc, Nodularia,* and *Nodosilenea,* 3.2–0.9, 3.2–0.1, and 3.2–0.3% respectively) were more abundant in the summer (Fig. 4).

Furthermore, sites H3 and H4 tended to be more homogeneous in terms of taxa abundance; particularly in site H3, bacteria belonging to the family Desulfosarcinaceae (1.9–4.1%) were more abundant in winter, yet bacteria belonging to the genus *Sulfurimonas* (3.3–0.2%) and *Truepera* (1.2–0.2%) dominated during the summer months. Finally, in H4 the most notable differences were in the abundances of the organisms identified as *Rhodohalobacter* (1.6–8.1%)*, Desulfotignum* (0.3–6.8%) during winter, and *Defluviicoccus* (2.8–0%) and *Chloroflexi* (0–2.5%) in summer. These results suggest that bacterial communities from Salar de Huasco change inter-seasonally in terms of diversity, abundance, and composition, as influenced by salinity, pH, and arsenic concentrations that could be directly affected by seasonal variations and water availability (Fig. 4).

**Fig. 5:**
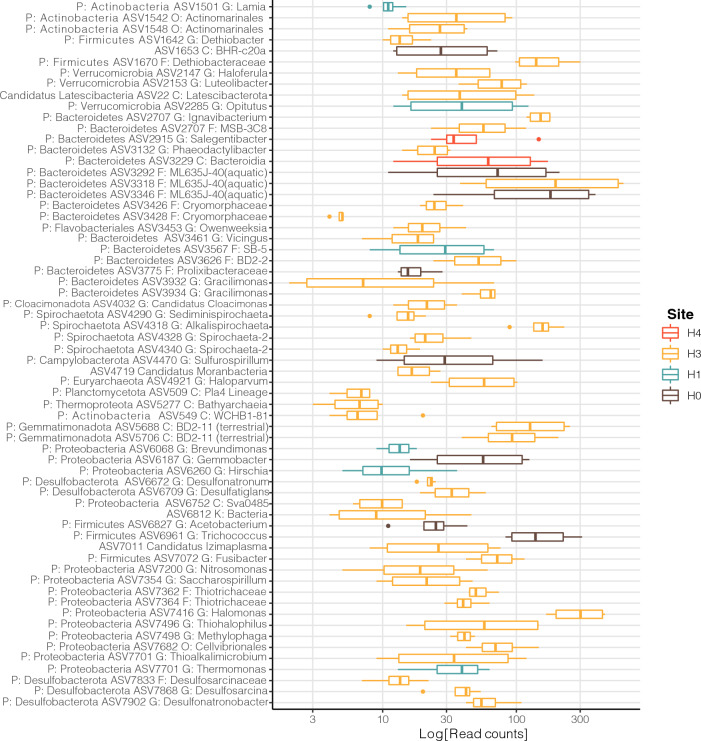
Relative abundance (log-transformed) of indicator species per site. Dots represent outliers, and box plots represent the interquartile range of the relative abundance tested using a permutation test, keeping biomarkers with a *P*-value ≤ 0.01. ASVs were assigned to the best taxonomic level available. Taxonomic ranks ‘phylum’ (P), ‘class’ (C), ‘order’ (O), or ‘family’ (F) are indicated when no genus-level (G) taxonomic assignment was available

Additionally, we performed an indicator species analysis to determine ASVs strongly associated with each site (Fig. 5), as previous results indicate that variations of sites within the wetland were significant [8, 9, 11]. We found 64 ASVs associated with the four sites, with the H3 site containing most of the indicator ASVs (48 ASVs), followed by the H0 site (8 ASVs), the H1 site (6 ASVs), and the H4 site with the least (2 ASVs).

For the H0 site, the eight ASVs were grouped into the Bacteroidetes (Bacteroidota), the Firmicutes (Bacillota), the Proteobacteria (Pseudomonadota)*,* and the Campylobacterota phyla. For site H1, the six ASVs were grouped in the Bacteriodetes (Bacteroidota), the Proteobacteria (Pseudomonadota), the Verrucomicrobiota (Verrucomicrobiota)*,* and the Actinobacteria (Actinobacteriota) phyla. At site H3, the 48 ASVs were grouped into 15 phyla including Actinobacteria (Actinobacteriota), Firmicutes (Bacillota), Verrucomicrobiota, and Proteobacteria (Pseudomonadota). Finally, the two ASV in site H4 were grouped inside the Bacteroidetes (Bacteroidota) phylum. There are some taxa that were found to be indicators that were also found to be among the most abundant taxa, such as *Luteolibacter*, *Halomonas,* and the ML635-J-40 family.

Subsequently, we predicted the metabolic potential of the indicator species using an approach based on phylogenetic placement (Supplementary Fig. S3). Samples were clustered in two clear groups, according to their metabolic potential, reflecting the same patterns observed for beta diversity and taxonomic composition. The grouping of H0 with H1, where reactions such as arsenate detoxification are abundant. Moreover, sites H3 with H4 were very similar, only little differences in glycogen and heme biosynthesis abundances were detected. However, there are some variations, namely the H0 site had higher relative abundance of myo-inositol and l-tryptophan degradation while H1 showed more enrichment of dichloroethane degradation metabolisms. In particular, the most abundant metabolic pathways were involved into redox reactions of formaldehyde, sulfate, sulfite, and octane; as well as in the degradation of toxic compounds (e.g., phenylmercury, 1,2-dichloroethane, and arsenate) and sugars (e.g., d-galactonate, d-arabinose, and d-sorbitol).

Overall, our results show that there are changes that are driven by small-scale variations, as differences appear to be geographically specific, with important influence of specific physicochemical parameters. Highlighting the necessity to study the enormous diversity harbored in natural laboratories such as poly-extreme environments found in the Chilean Altiplano, where highly adapted microorganisms thrive, however small variations can trigger loss of biodiversity that in these oligotrophic environments can be traduced in changes in the biogeological cycles.

## Discussion

Microbial communities that inhabit sediments in Salar de Huasco, a high-altitude wetland, are dynamic and influenced by small-scale geographical (site-dependent) and seasonal variations, mainly associated with parameters that vary according to water availability, as there are correlations of microbial diversity with pH, salinity, and arsenic concentrations. The sampled sites follow a natural and known arsenic gradient [26], and our findings coincide with previous reports of high taxonomic variability within the wetland [7–9, 11, 28]. There is also a known well-marked environmental cyclicity and defined wet and dry seasons [6].

Moreover, climate conditions influence these dynamics as evidenced by differences in diversity indexes, structure, abundance, and composition of the bacterial communities (Figs. 1, 2, 3 and 4). Rainfall directly determines water availability and the concentrations of dissolved elements, including nutrients, metal(oids) [13, 14], thus microbial communities in sediments are more responsive to temporal changes than those found in water. This impact is especially relevant in the Altiplano area, where summer months are associated with heavy rainfall episodes known as the “Bolivian winter,” while summertime precipitation is virtually the only water source on the South American Altiplano, a phenomenon that is closely related to the El Niño–Southern Oscillation (ENSO) [5].

Diversity indices show inter-seasonal changes in sites: H0, H1, and H4, while H3 showed no statistically significant change associated with the seasonal variable (Fig. 1). In SH, salinity increases from north to south, as determined by a model proposed by [12]. Thus, water and minerals are transported and gradually deposited as the water current loses strength and would therefore be more concentrated toward the sampled site H4 (Fig. 2). These support the influence of SH geochemical dynamics over the microbial communities, as this abiotic pressure triggers adaptation and selection of microbes with higher fitness, especially against osmotic stress.

Another relevant variable in shaping the community was arsenic, a metalloid found free in the environment accumulating in the form of two oxyanions As(III) and As(V). Therefore, its cycle, solubility, and bioavailability depend on physicochemical conditions, such as iron content, sulfates, redox potential, and pH [29, 30]. Particularly, pH is important as acidic values promote the dominance of As(V), while under alkaline pH conditions, As(III) is more prevalent [31]; which is relevant since all the pH values reported here for SH vary within the alkaline range (Table 1). Consequently, a larger proportion of the more-toxic As(III) would be expected, thus exerting a strong pressure on the bacterial communities, especially during the winter; when the total arsenic values were higher in all four sites. Thus, it would be expected to observe a high variety of microorganisms capable of metabolizing arsenic, through oxide-reduction and methylation reactions [32], as evidenced in this study in sites H3 and H4 (Fig. S3).

Additionally, beta-diversity analysis shows that sites rather than seasons seem to drive species turnover (Fig. 2), as previously reported there is a great variation among SH sites [9, 11], but there is also cyclicity associated with broader climatic phenomena, where clear dry and wet periods are appreciated [6], therefore the results found in this study further contribute to the understanding of dynamic changes that occur within this poly-extreme wetland that are subjected to niche-specific changes and can be evidenced in a small geographical scale.

Although there are no overall significant differences (within all pairwise comparisons) associated with seasonality, there are evident changes in taxa abundance, distribution, and presence in three sites associated with changes between the wet and dry periods (Fig. 3). In accordance with previous taxonomic characterizations in Salar de Huasco the community is dominated by Proteobacteria and Bacteroidetes [7–9, 11]. Yet, there is a great proportion of ASVs that could not be classified to low taxonomic ranks in these extreme bacterial communities, that might be active participants in maintaining a functioning ecosystem, which demonstrates how little explored these environments continue to be and how the database shortage continues to be a major block for research. While Proteobacteria displays a different dynamic for each site, Bacteroidota increases during the winter season, and together they represent from 36 to 68% of all classified reads. There are observed changes in the abundance of some taxa (Figs. 3 and 4), that are significantly different (Wald Test: *P* ≤ 0.01, Supplementary Table S1), and sites H0, H1 and H4 show statistically significant changes in alpha diversity between the wet and dry periods (Fig. 1).

Particularly, for the H0 site, the results at the phylum level indicate that the greater inter-season variations occur in unclassified members of Cyanobacteria, that could be specialized to these conditions as this group of microorganisms is composed of primary producers that can take advantage of the high radiation and some species are capable of diazotrophic growth and play an important role in biogeochemical processes [33]. Also, there is evidence showing salinity and arsenic relevance in shaping microbial communities [34, 35].

The bacterial community from site H1 shows more diversity during winter; additionally, we detected an increase in abundance of less common phyla. Previously it was determined that the H1 community profile possesses high abundance of Chloroflexi, Actinobacteria, Verrucomicrobia, Planctomycetes, and Acidobacteria phyla [11]. However, in the present study, Chloroflexi, Desulfobacterota, and Proteobacteria are the phyla with greatest inter-season variations. Chloroflexi comprises extremophiles that are metabolically diverse, including clades capable of anoxygenic phototrophy or fix inorganic CO_2_ [36], a metabolic diversity that is relevant in this oligotrophic environments where nutrient acquisition and organic transformation are vital for maintaining the entire ecosystem.

The sulfate reducing Desulfobacterota are known to be involved in iron metabolism, thiosulfate and tetrathionate reduction, carotenoid biosynthesis, and fermentation [37], therefore changes in abundance, along with members of Proteobacteria*,* could be a consequence of geochemical alterations associated with changes in seasons, namely metal(oids) solubilization and water availability.

To further characterize the bacterial community, the most abundant taxa (ASV) at the lowest available taxonomic rank were identified (Fig. 4). The bacterial composition is different in each season; while some taxa remain stable, others display marked differences and even disappear in some cases. For example, the most drastic change in site H0 was that of an unknown species of Cyanobacteria that represented 7% of the total classified reads in winter yet was completely absent in the summer. These phyla encompass autotroph, heterotroph, or mixotroph microorganisms able to grow in high temperatures, low water potential, and high radiation. They are also great colonizers, as they require a short-wet period for metabolic activation enhancing the uptake of essential nutrients [38]. There are also changes that could be associated with nutrient availability, for instance during summer there were no detection of the sulfur-oxidizer chemolithoautotroph *Sulfurovum,* and *Bacteroides* while it represented 4.7% and 4.5% of the community during winter.

Also, in site H1, the most significant variation occurs in the abundance of *Defluviicoccus* (9.8% to 0.1% from summer to winter), this genus is associated with glycogen accumulation [39] that could be key in the energy dynamics of the community. Finally, in H4, *Rhodohalobacter,* a facultative anaerobic and halophilic microorganism which is the most abundant taxa of the study (only detected in H3 and H4) showed the biggest difference between seasons, together with an unknown Cyanobacteria, taxonomic groups that are closely dependent on water availability and salinity concentrations.

To further study the dynamics that each geo-chemical conditions influenced upon the microbial communities, we determined the indicator species for each sampled site (Fig. 5), the findings reveal that the site H3, shown to be the most stable, displayed the greater number of indicator species, suggesting a broader plethora of taxa and potential metabolisms that can maintain a homogeneous community. Whereas the site H4 that showed seasonal changes in alpha diversity (regardless the used index) presented only one ENS phyla (with two indicator species), including an aerobic, non-motile halotolerant bacteria [40].

Additionally, sites H1 and H0 encompass their indicator species among 4 phyla. In particular, site H0 included psychrotolerant, anaerobic, aerobic, chemoorganotrophic taxa (*Trichococcus*, *Acetobacterium*, *Gemmobacter*) as well as the sulfur, arsenate, and tetrachloroethane reducing *Sulfurospirilim* [41, 42]. On the other hand, site H1 indicator species include aerobic, anaerobic, nitrite-reducing, and cosmopolitan taxa such as *Brevundimonas*, *Thermomonas,* and *Salegentibacter* among others [43, 44]. Therefore, the specific dynamics in metabolic services could be a determining factor in the microbial communities found in the sediments of SH. However, this approximation must be complemented in future studies that take into consideration strain variability within the species and experimental validation of the microbial functions.

Overall, the characterization of the specific changes in the bacterial community composition suggests a high diversity of metabolic and niche-specific adaptations that allow these microorganisms to thrive and be successful in an extreme and dynamic environment. In particular, the analysis shows that the microbial community that inhabits site H0 is enriched in metabolic functions that maintain central carbon metabolism and growth in limited nutrient conditions, such as phosphate acquisition, D-arabinose degradation, l-idonate degradation among others. Furthermore, in H1 functions associated with glycogen biosynthesis and taurine degradation are primarily found, suggesting differences in energy supply during growth-limiting conditions [45] also detoxifying pathways seem to the critical, such as phenylmercury acetate degradation, linked to the rich geochemical nature of the wetland [46], thus reflecting the nature of the chemical availability in each site.

Also, in sites H4 and H3 the most enriched predicted metabolisms are associated with arsenate detoxification, as metalloids concentrations could be increased by deposition in this area of the Salar. This predictive analysis suggests that in site H3 the microbial community uses degradation of sulfur compounds as energy sources [47], also to cope with osmotic stress microorganisms found in this site seem to be synthesizing amino acid derivatives such as ectoine [48]. Finally, membrane cell biosynthesis, in the form of sphingolipids [49], and acid stress response [50] seem to be the more relevant functions in the communities that inhabit site H4. The metabolic predictions contribute to the hypothesis of the highly dynamic aspects of the microbial life in Salar de Huasco, where specialization can be a critical feature for survival in the poly-extreme environment.

These results contribute to the knowledge of the mechanisms that maintain this fragile ecosystem, where geographically close sites have different physicochemical and microbial communities, therefore changes in the diversity, structure, and composition of the shallow sediment bacterial community are driven by small-scale changes in geo-chemical characteristics such as arsenic and salt concentrations, pH influenced by seasonal variations which can affect each particular sampled site differently. Broader studies including the use of geochemical markers, and an in-depth analysis of the dynamics of the biota and the abiotic parameters must be considered to further understand the dynamics that drive the great microbial diversity that is highly adapted but can be easily disturbed by climate changes and the mining industry.

## Conclusion

The bacterial communities in Salar de Huasco are highly diverse and are susceptible to small-scale changes in geography as well as variations in seasonality, in particular water availability. In this study we evidenced changes in diversity associated with site collection and summer/winter seasons. Also, diversity is driven mainly by pH values, arsenic and NaCl concentrations, all parameters that are strongly related to the amount of rainfall and dissolved solutes. In terms of abundance important groups such as Cyanobacteria, Proteobacteria, and Bacteriodota are among the most abundant as well as the taxa that presents variations in seasonality. Finally, each site has a particular set of parameters that drive the composition of the community, including the indicator species, suggesting that the high diversity of the ecosystem is associated with different specialized communities that thrive in a relatively small geographical area.

## Supplementary Information

Below is the link to the electronic supplementary material.

Supplementary Figure S1 Map of the study area: Salar de Huasco. Map showing the four sampling sites investigated in this study (H0: 20°15948.899S, 68°52928.499W; H1: 20°16927.799S, 68°539399W; H3: 20°16959.299S, 68°53916.799W and H4: 20°17940.999S, 68°53917.399W). The SH is located between 68°479, 68°549 W and 20°159, 20°209 S in the Tarapacá region of northern Chile (Google Earth) (PDF 6223 KB)

Supplementary Figure S2. Taxonomic composition and relative abundance of the microbial communities in the four studied sites in Salar de Huasco (H0, H1, H3, H4); stacked bars show the 20 most abundant bacteria at phylum rank (PDF 152 KB)

Supplementary Figure S3 Relative abundance of metabolic pathways prediction for indicator species. The prediction was made based on the phylogenetic placement of the ASVs. The relative abundance of each metabolic pathway was inferred from the reads associated with the indicator species. The colors of the heat map represent the relative abundance (log-transformed) of the metabolic pathways (PDF 13 KB)

Supplementary file4 (DOCX 15 KB)

Supplementary file5 (DOCX 16 KB)

## Data Availability

The raw sequencing data presented in this study have been deposited in the DDBJ/ENA/GenBank SRA database under the BioProject: PRJNA573913.
