# Supplementary figures and images for: Exploring the Influence of Small-Scale Geographical and Seasonal Variations Over the Microbial Diversity in a Poly-extreme Athalosaline Wetland
Source: Curr Microbiol. 2023 Jul 25;80(9):297. doi: 10.1007/s00284-023-03395-w (PMC10368551; doi:10.1007/s00284-023-03395-w)

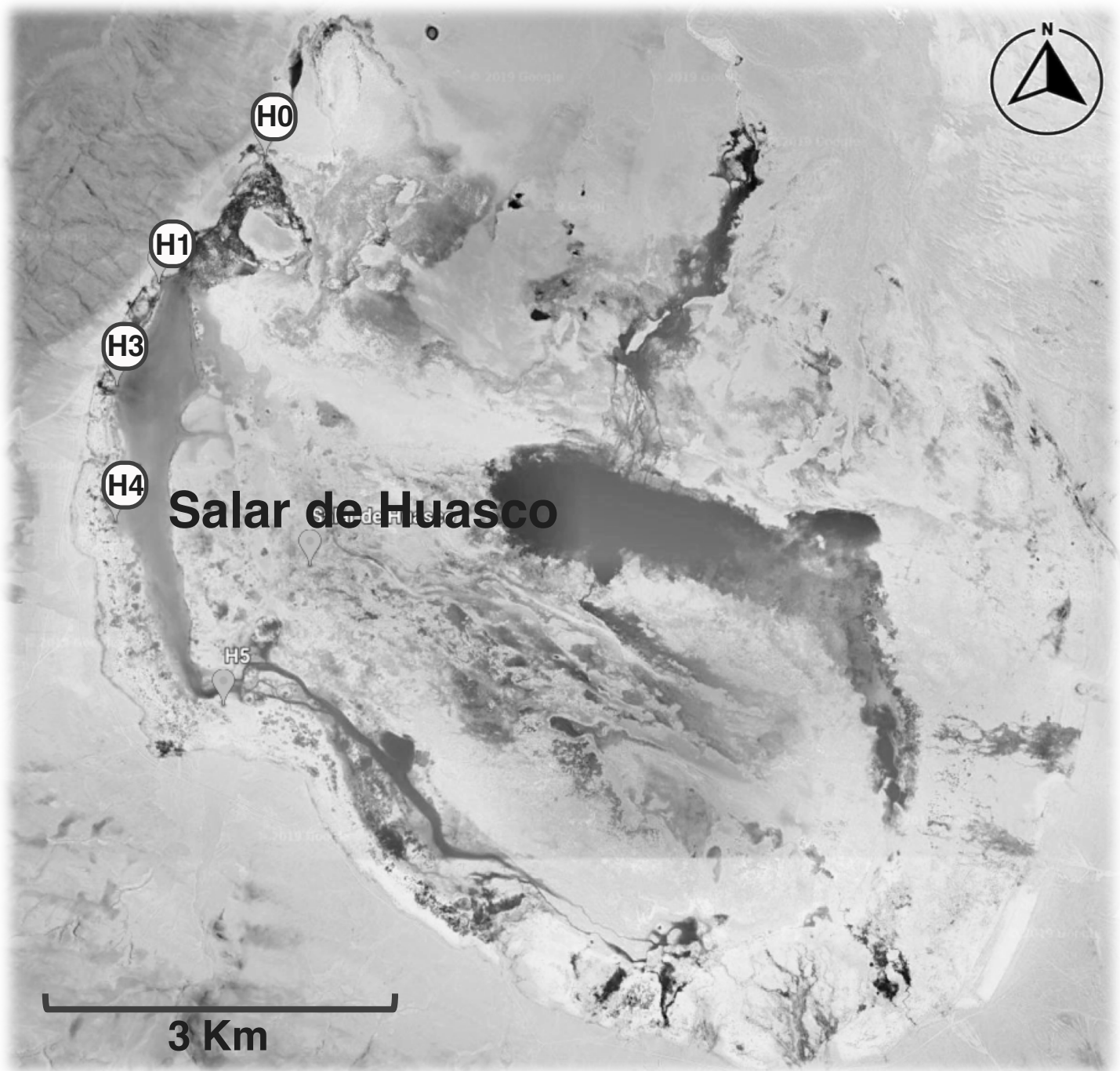

Supplement: Supplementary file 1 — Supplementary Figure S1 Map of the study area: Salar de Huasco. Map showing the four sampling sites investigated in this study (H0: 20°15948.899S, 68°52928.499W; H1: 20°16927.799S, 68°539399W; H3: 20°16959.299S, 68°53916.799W and H4: 20°17940.999S, 68°53917.399W). The SH is located between 68°479, 68°549 W and 20°159, 20°209 S in the Tarapacá region of northern Chile (Google Earth) (PDF 6223 KB) [file 284_2023_3395_MOESM1_ESM.pdf]

# Summer

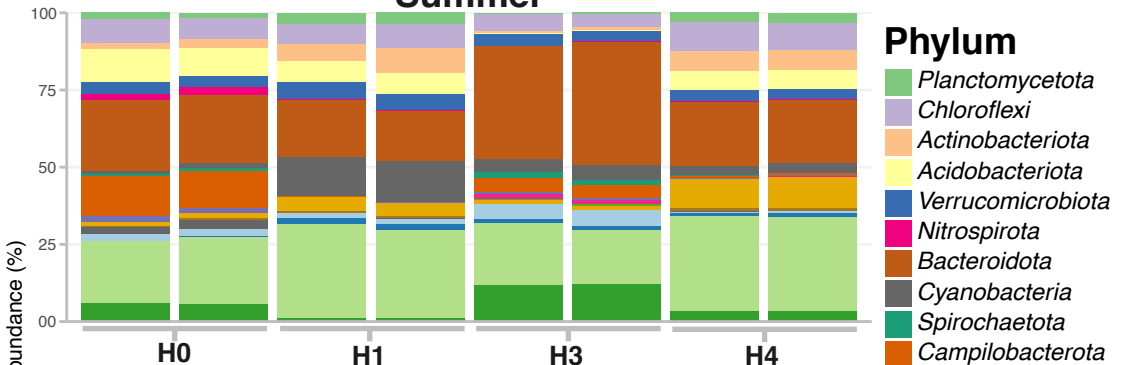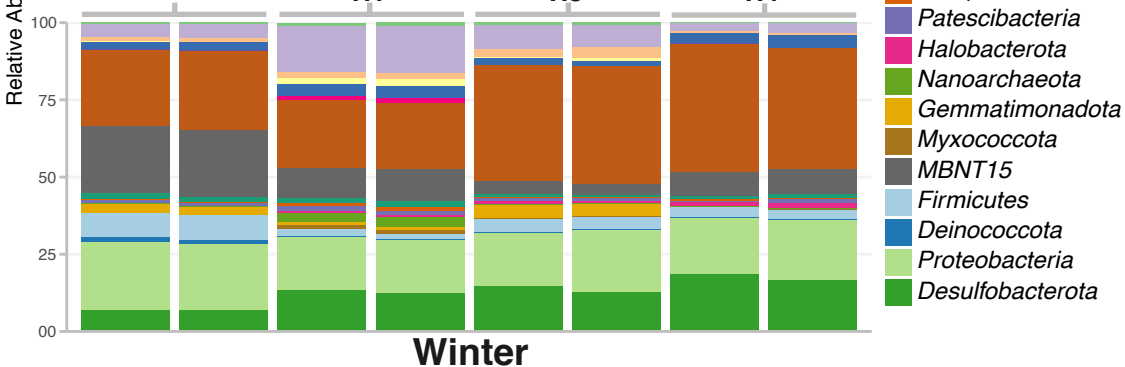

Supplement: Supplementary file 2 — Supplementary Figure S2. Taxonomic composition and relative abundance of the microbial communities in the four studied sites in Salar de Huasco (H0, H1, H3, H4); stacked bars show the 20 most abundant bacteria at phylum rank (PDF 152 KB) [file 284_2023_3395_MOESM2_ESM.pdf]

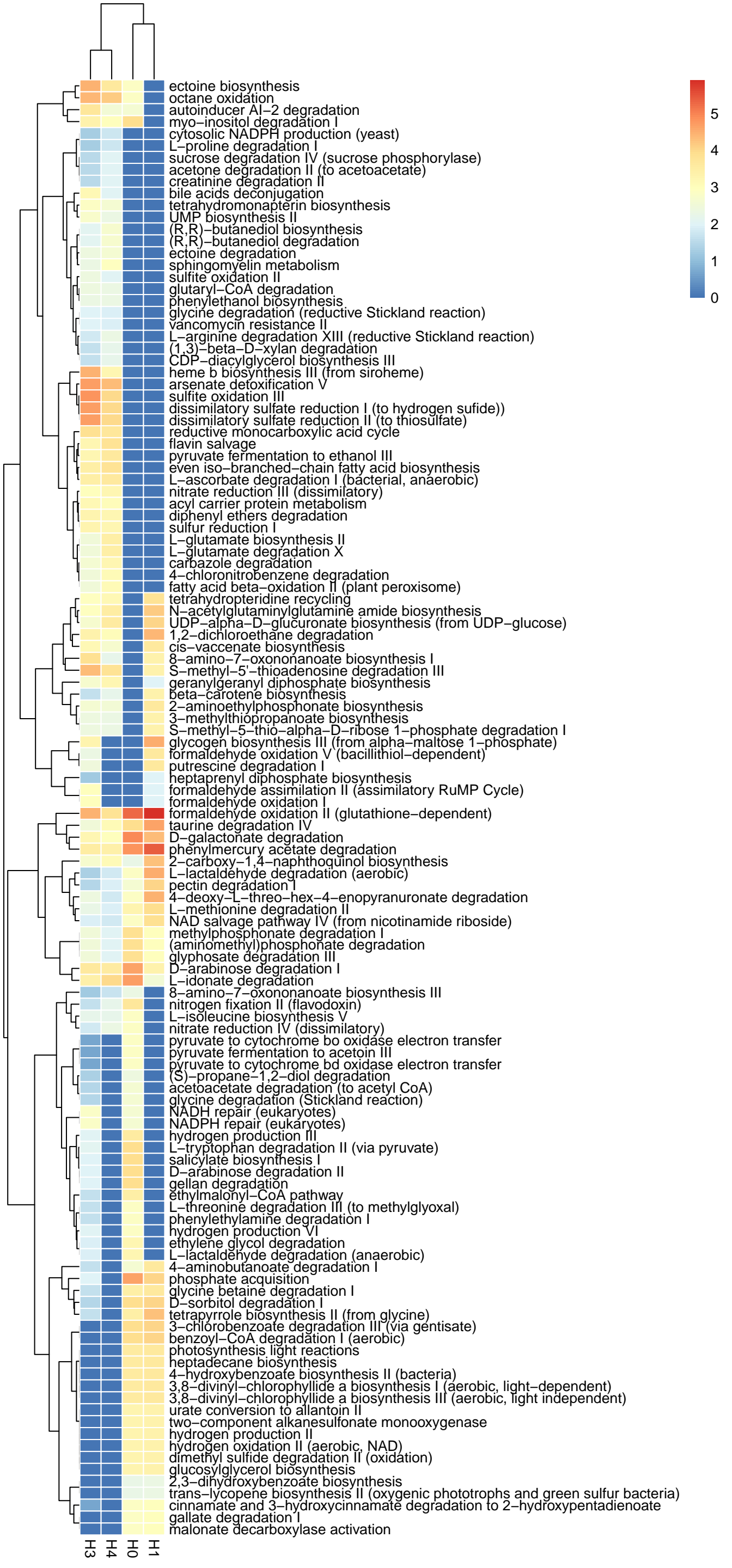

Supplement: Supplementary file 3 — Supplementary Figure S3 Relative abundance of metabolic pathways prediction for indicator species. The prediction was made based on the phylogenetic placement of the ASVs. The relative abundance of each metabolic pathway was inferred from the reads associated with the indicator species. The colors of the heat map represent the relative abundance (log-transformed) of the metabolic pathways (PDF 13 KB) [file 284_2023_3395_MOESM3_ESM.pdf]
