# Supplementary material for: Exploring the Influence of Small-Scale Geographical and Seasonal Variations Over the Microbial Diversity in a Poly-extreme Athalosaline Wetland
Source: Curr Microbiol. 2023 Jul 25;80(9):297. doi: 10.1007/s00284-023-03395-w (PMC10368551; doi:10.1007/s00284-023-03395-w)
Supplement: Supplementary file 4 — Supplementary file4 (DOCX 15 KB) [file 284_2023_3395_MOESM4_ESM.docx]

| **Supplementary Table S1.** Wald Test adjusted *p*-values for differential abundance comparison of the top 30 phyla in the four SH sites between both seasons. | | | | |
| --- | --- | --- | --- | --- |
| **Phylum** | **H0** | **H1** | **H3** | **H4** |
| Acidobacteriota | 0.0000000 | 0.000000 | 0.0917197 | 0.000000 |
| Actinobacteriota | 0.2662382 | 0.000000 | 0.0000000 | 0.000000 |
| Armatimonadota | 0.2119417 | 0.060388 | 0.0217337 | 1.000000 |
| Bacteroidota | 0.0000000 | 0.025727 | 0.0019074 | 0.000000 |
| Bdellovibrionota | 0.2176426 | 0.000803 | 0.0051796 | 0.000499 |
| Campilobacterota | 0.0000000 | 0.000000 | 0.0000000 | 0.206895 |
| Chloroflexi | 0.8909010 | 0.005320 | 0.2720975 | 0.000000 |
| Cloacimonadota | 0.0000000 | 0.000000 | 0.0000221 | 0.000487 |
| Crenarchaeota | 0.0000001 | 0.000000 | 0.0000003 | 0.000000 |
| Cyanobacteria | 0.0000000 | 0.000000 | 0.0000003 | 0.000000 |
| Deinococcota | 0.0000000 | 0.000000 | 0.0000000 | 0.001683 |
| Desulfobacterota | 0.0000000 | 0.000000 | 0.0674527 | 0.000000 |
| Fibrobacterota | 0.4303294 | 0.076293 | 0.1521722 | 0.785365 |
| Firmicutes | 0.0000000 | 0.003764 | 0.0000002 | 0.000000 |
| Gemmatimonadota | 0.0000000 | 0.000000 | 0.0000000 | 0.000000 |
| Halanaerobiaeota | 0.1066813 | 0.000034 | 0.0000000 | 0.000000 |
| Halobacterota | 0.0000000 | 0.000000 | 0.0000374 | 0.000000 |
| Latescibacterota | 0.0000008 | 0.000000 | 0.0167648 | 0.495873 |
| LCP-89 | 0.0001454 | 0.000003 | 0.0000000 | 0.000000 |
| MBNT15 | 0.0000000 | 0.374009 | 0.0009858 | 0.000001 |
| Methylomirabilota | 0.1056139 | 0.762288 | 1.0000000 | 1.000000 |
| Myxococcota | 0.1588789 | 0.762288 | 0.0000000 | 0.000000 |
| Nanoarchaeota | 0.5874855 | 0.000000 | 0.0000000 | 0.000000 |
| NB1-j | 0.0000003 | 0.000039 | 0.0044490 | 0.000058 |
| Nitrospirota | 0.0000000 | 0.000000 | 0.0000000 | 0.000000 |
| Other | 0.8377630 | 0.000000 | 0.0004669 | 0.608080 |
| Patescibacteria | 0.9495357 | 0.000000 | 0.4738115 | 0.000000 |
| Planctomycetota | 0.0000096 | 0.000000 | 0.0251726 | 0.000000 |
| Proteobacteria | 0.0000000 | 0.000000 | 0.0015762 | 0.001091 |
| Spirochaetota | 0.0000000 | 0.000000 | 0.0000000 | 0.000000 |
| Verrucomicrobiota | 0.1092346 | 0.000000 | 0.0000000 | 0.002017 |
