# Supplementary material for: Exploring the Influence of Small-Scale Geographical and Seasonal Variations Over the Microbial Diversity in a Poly-extreme Athalosaline Wetland
Source: Curr Microbiol. 2023 Jul 25;80(9):297. doi: 10.1007/s00284-023-03395-w (PMC10368551; doi:10.1007/s00284-023-03395-w)
Supplement: Supplementary file 5 — Supplementary file5 (DOCX 16 KB) [file 284_2023_3395_MOESM5_ESM.docx]

| **Supplementary Table S2.** Wald Test adjusted *p*-values for differential abundance comparison of the top 30 taxa in the four SH sites between both seasons. | | | | | | |
| --- | --- | --- | --- | --- | --- | --- |
| **ASV** | **Phylum** | **Lowest-rank** | **H0** | **H1** | **H3** | **H4** |
| ASV3945 | Bacteroidota | Rhodohalobacter | 1.0000000 | 1.0000000 | 0.2268245 | 0.0081181 |
| ASV7821 | Desulfobacterota | Desulfotignum | 1.0000000 | 1.0000000 | 1.0000000 | 0.0000000 |
| ASV4201 | Cyanobacteria | Chloroplast | 0.0000004 | 0.0000000 | 0.0093945 | 0.1494619 |
| ASV6345 | Proteobacteria | Defluviicoccus | NA | 0.0001672 | 0.0137402 | 0.0169437 |
| ASV7425 | Proteobacteria | Marinobacter | 1.0000000 | 1.0000000 | 0.0055921 | 0.0000000 |
| ASV4191 | Cyanobacteria | Chloroplast | 0.6345702 | 1.0000000 | 0.0010014 | 0.0002478 |
| ASV772 | Chloroflexi | ADurb.Bin120 | NA | 1.0000000 | 0.0139010 | 0.0000000 |
| ASV6198 | Proteobacteria | Roseovarius | NA | 1.0000000 | 0.0640217 | 0.0000873 |
| ASV7839 | Desulfobacterota | Desulfosarciceae | 0.0000000 | 0.1657866 | 0.0000000 | 0.5110439 |
| ASV2117 | Verrucomicrobiota | Luteolibacter | 0.8382114 | 1.0000000 | 0.0260774 | 1.0000000 |
| ASV4205 | Cyanobacteria | Chloroplast | 0.0000005 | 0.0000009 | 0.3777076 | 1.0000000 |
| ASV7189 | Proteobacteria | Thiobacillus | 0.0000000 | 0.0000001 | 0.2002142 | 0.0000000 |
| ASV4466 | Campilobacterota | Sulfurimos | 0.0035892 | 1.0000000 | 0.0078200 | 1.0000000 |
| ASV3765 | Bacteroidota | Prolixibacteraceae | 1.0000000 | 1.0000000 | 1.0000000 | 0.3463700 |
| ASV2698 | Bacteroidota | Ignavibacterium | 0.0272896 | 0.7323000 | 0.0348766 | 0.9234500 |
| ASV3335 | Bacteroidota | ML635J-40_aquatic_group | 1.0000000 | 1.0000000 | 0.0010528 | 0.0000000 |
| ASV7124 | Deinococcota | Truepera | 1.0000000 | 0.0037429 | 1.0000000 | 0.0723100 |
| ASV7407 | Proteobacteria | Halomonas | 0.4175077 | 0.3208673 | 0.0072377 | 0.0000000 |
| ASV4453 | Campilobacterota | Sulfurovum | 0.0358919 | 1.0000000 | 1.0000000 | 1.0000000 |
| ASV7251 | Proteobacteria | Hydrogenophaga | 0.0794653 | 0.0020923 | 0.0781410 | 0.0727571 |
| ASV3956 | Bacteroidota | Balneolaceae | 1.0000000 | 1.0000000 | 0.0833286 | 0.0000000 |
| ASV3009 | Bacteroidota | Bacteroides | 0.0318916 | 1.0000000 | 1.0000000 | 1.0000000 |
| ASV4244 | Cyanobacteria | Nodularia_PCC-9350 | NA | 0.0000000 | 1.0000000 | 1.0000000 |
| ASV4231 | Cyanobacteria | Nostoc_PCC-73102 | NA | 1.0000000 | 0.0087421 | 1.0000000 |
| ASV4139 | Cyanobacteria | Nodosilinea_PCC-7104 | 0.1427496 | 0.0003476 | 1.0000000 | 0.0741965 |
| ASV3947 | Bacteroidota | Balneolaceae | 1.0000000 | 1.0000000 | 0.1644096 | 0.2590075 |
| ASV3894 | Bacteroidota | Algoriphagus | 1.0000000 | 0.1390102 | 0.3278000 | 1.0000000 |
| ASV911 | Chloroflexi | Aerolineaceae | 1.0000000 | 0.1865069 | 0.2307536 | 0.0000000 |
| ASV3356 | Bacteroidota | ML635J-40_aquatic_group | 0.0322423 | 1.0000000 | 0.8718132 | 0.4727324 |
| ASV6226 | Proteobacteria | Roseibaca | 0.9754718 | 1.0000000 | 0.8718132 | 0.0169437 |
